# Spatial-Temporal Signals and Clinical Indices in Electrocardiographic Imaging (II): Electrogram Clustering and T-Wave Alternans

**DOI:** 10.3390/s20113070

**Published:** 2020-05-29

**Authors:** Raúl Caulier-Cisterna, Manuel Blanco-Velasco, Rebeca Goya-Esteban, Sergio Muñoz-Romero, Margarita Sanromán-Junquera, Arcadi García-Alberola, José Luis Rojo-Álvarez

**Affiliations:** 1 Department of Signal Theory and Communications, Telematics and Computing Systems, Rey Juan Carlos University, 28943 Fuenlabrada, Madrid, Spain; raul.caulier@urjc.es (R.C.-C.); rebeca.goyaesteban@urjc.es (R.G.-E.); sergio.munoz@urjc.es (S.M.-R.); margarita.sanroman@urjc.es (M.S.-J.); 2 Department of Signal Theory and Communications, Universidad de Alcalá, 28805 Alcalá de Henares, Madrid, Spain; manuel.blanco@uah.es; 3 Center for Computational Simulation, Universidad Politécnica de Madrid, 28223 Boadilla, Madrid, Spain; 4 Arrhythmia Unit, Hospital Clínico Universitario Virgen de la Arrixaca de Murcia, El Palmar, 30120 Murcia, Spain; arcadi@secardiologia.es

**Keywords:** Electrocardiographic Imaging, signal processing, cardiac indices, spatial–temporal processing, Long-QT syndrome, infarction, T-wave alternans, control subject

## Abstract

During the last years, attention and controversy have been present for the first commercially available equipment being used in Electrocardiographic Imaging (ECGI), a new cardiac diagnostic tool which opens up a new field of diagnostic possibilities. Previous knowledge and criteria of cardiologists using intracardiac Electrograms (EGM) should be revisited from the newly available spatial–temporal potentials, and digital signal processing should be readapted to this new data structure. Aiming to contribute to the usefulness of ECGI recordings in the current knowledge and methods of cardiac electrophysiology, we previously presented two results: First, spatial consistency can be observed even for very basic cardiac signal processing stages (such as baseline wander and low-pass filtering); second, useful bipolar EGMs can be obtained by a digital processing operator searching for the maximum amplitude and including a time delay. In addition, this work aims to demonstrate the functionality of ECGI for cardiac electrophysiology from a twofold view, namely, through the analysis of the EGM waveforms, and by studying the ventricular repolarization properties. The former is scrutinized in terms of the clustering properties of the unipolar an bipolar EGM waveforms, in control and myocardial infarction subjects, and the latter is analyzed using the properties of T-wave alternans (TWA) in control and in Long-QT syndrome (LQTS) example subjects. Clustered regions of the EGMs were spatially consistent and congruent with the presence of infarcted tissue in unipolar EGMs, and bipolar EGMs with adequate signal processing operators hold this consistency and yielded a larger, yet moderate, number of spatial–temporal regions. TWA was not present in control compared with an LQTS subject in terms of the estimated alternans amplitude from the unipolar EGMs, however, higher spatial–temporal variation was present in LQTS torso and epicardium measurements, which was consistent through three different methods of alternans estimation. We conclude that spatial–temporal analysis of EGMs in ECGI will pave the way towards enhanced usefulness in the clinical practice, so that atomic signal processing approach should be conveniently revisited to be able to deal with the great amount of information that ECGI conveys for the clinician.

## 1. Introduction

Cardiac arrhythmias are still one of the leading prevalence illnesses in our days, and they consist either of alteration in the origin of the bioelectrical impulses controlling the normal rhythm of the heart, or alterations in the propagation paths for these impulses, or in alterations in both of them. Whereas the Electrocardiogram (ECG) is the main diagnostic tool for cardiac arrhythmias, the recently proposed Electrocardiographic Imaging (ECGI) systems are opening new ways towards arrhythmia diagnosis [1,2,3]. Attention and effort is being devoted to EGCI in the last years, as far as first commercially available equipments are being used in the Cardiac Electrophysiology Labs [4,5,6,7].

Together with the raise of ECGI systems used in practice, technical sides have been also analyzed lately, in terms of potential estimation and signal processing. A comprehensive review was presented in [8] for the current dipole (vectorcardiogram), the equivalent double layer, and the potential transfer direct and inverse problem formulations. In addition, a closed-form expression was delivered for potential fields generated by triangular monolayers with linear distributions [8]. These works can be seen as references, among many others, for the biophysics of the problem to be taken into account. When bringing ECGI to the clinical practice, it has been evident that not only the accuracy of the inversion methods from the torso potential matters, but also the signal preprocessing and the connection of the retrieved information with the information conventionally used by the electrophysiologists need to be further harmonized. As an example, a review on the scope of Cardioinsight${}^{TM}$ system [3] pointed out that estimated cardiac potentials should be checked manually to avoid errors from processing. In [9], it is suggested that advances on real-time panoramic mapping and signal processing are emerging, but some issues persist, like the endocardial sequence of activation not being equal to the epicardial, or the septum not being an epicardial structure, or suboptimal detection of arrhythmia activation from corresponding chamber. In [10], a nearest-neighbor algorithm was used to avoid map smoothing, between-map correlation was documented to be sometimes low and with high interpatient variability, and lines of block and epicardial breakthrough sites were inaccurately imaged in several cases. In [11], thoughts are shared on the possible contribution of these systems to atrial fibrillation ablation, as well as on attempts to date to apply therapeutic strategies based on incomplete understanding of mechanisms, like fragmented electrograms (EGM), rotors, or focal sources. The same authors point the agreement that the wavefront complexity in many arrhythmia mechanisms is characterized by functional wavefront blocks possibly facilitated by spatial dispersion of refractoriness and intra-atrial differences in fibrosis, and all of this should be possibly visualized and scrutinized with advantage by using ECGI systems. The Consortium for Electrocardiographic Imaging (CEI) aims to facilitate the collaboration across the community and to build standards and benchmarking setups, as stated in some recently delivered work [12].

Overall, a new generation of possibilities are to be scrutinized in the following years aiming to connect the previously used intracardiac EGM acquired during electrophysiological studies in the diagnosis and treatment of arrhythmias. From the previous and other contributions we can envision that spatial–temporal characteristics of digital signal processing for cardiac biopotentials should now be analyzed from a new perspective. Aiming to contribute to the usefulness of ECGI recordings in the current knowledge and methods of cardiac electrophysiology, we scrutinized two main results in the companion paper [13]: First, spacial consistency can be accounted and observed even for very basic cardiac signal processing stages (baseline wander, low-pass filtering); second, bipolar EGMs can be obtained by an operator searching for the maximum amplitude and including a time delay. In this work, spatial consistency is tackled from a twofold viewpoint: first, by studying the ECG waveforms, and second, by studying the ventricular repolarization properties. For the first case, and in order to find spatial coherence among waveforms, a regionalization study is addressed in terms of the clustering properties of the unipolar and bipolar EGM waveforms, both in control and in myocardial infarction patients. On the other hand, the ventricular repolarization is analyzed through the properties of T-wave alternans (TWA) in control and in Long-QT syndrome (LQTS) subjects. T-wave alternans is a noninvasive predictor of malignant arrhythmias, which has been shown to be a a good indicator for stratifying risk of sudden cardiac death [14,15,16]. In the present work we address the assessment of TWA in ECGI recordings to characterize the test with regionalization and spatial specificity, hence identifying those cardiac regions with functional cardiac instability according to this marker.

The scheme of the paper is as follows. The fundamentals of clustering analysis, together with the existing methods for TWA estimation in ECG signals, are first summarized, together with the dataset description. Experiments are driven for analyzing the EGM regionalization and the TWA spatial–temporal distribution of the proposed alternans markers. Conclusions are finally drawn.

## 2. Methods and Materials

The interest of this paper is the study of consistency of ECGI techniques, which is tackled here from two viewpoints. First, EGM waveform morphology is analyzed in global terms by seeking to the joint signal similarities existing throughout the whole cardiac tissue. This is addressed by using clustering techniques to separate coherent myocardial areas using both unipolar and bipolar EGMs from different patients; thus, an introduction of clustering methods is provided in this section. Second, the analysis of the diagnostic information content embedded in the signals and its distribution throughout the heart is faced by studing the phenomenon of repolarization alternans. For this purpose, three representative methods for TWA characterization are chosen and briefly introduced.

Regarding the database, both the fundamentals of ECGI and the description of the dataset are introduced in the companion paper, so we refer the reader to that work for deeper details [13]. In brief, a body surface map of bioelectrical potentials taken on the torso of a patient, along with his/her heart-torso geometry data, is used to derive the epicardial activity obtained as the solution of the inverse problem in electrocardiography by means of the so called ECGI algorithms. For this work, we were provided with a dataset from Yoram Rudy Lab at Washington University in St. Louis containing both body surface potential mapping, and epicardial meshes from control and patients suffering different pathologies [2,17,18,19], among which, several of them were chosen to conduct the experiments as detailed further. The acquired data consisted of 205 and 228 useful recording electrodes (for the LQTS and control subjects, respectively) from the BSPM system on the torso, and they were used to generate 502 and 1002 estimated potentials on the heart epicardium.

Notice that from ECGI, the available information is provided in terms of unipolar potentials. The notation to describe unipolar EGMs in a surface mesh point, though not necessarily needed here, is presented in the companion paper. Nonetheless, in clinical routine, information provided by bipolar potentials is used to heal and diagnosing diseases during Electrophysiological Study (EPS), so in order to make bipolar EGMs available to clinicians, these kind of EGMs are computed through the application of signal processing methods to the original unipolar EGMs. In this work, bipolar potentials at one point ${\bar{s}}_{i}$ of the surface mesh, representing the cardiac tissue, is determined as follows:

(1) $$\beta \left({\bar{s}}_{i},t_{w}\right)=v\left({\bar{s}}_{i},t_{w}\right)-v\left({\bar{s}}_{k},\left(t_{w}-\alpha \right)\right),$$

where $v({\bar{s}}_{i},t_{w})$ is the unipolar potential at the node ${\bar{s}}_{i}$ for a time window duration of $t_{w}$, and $v\left({\bar{s}}_{k},\left(t_{w}-\alpha \right)\right)$ is the unipolar potential at the node ${\bar{s}}_{k}$ for the same time window. The node ${\bar{s}}_{k}$ is chosen among the adjacent ones of ${\bar{s}}_{i}$ according to some specific criterion. In addition, a time delay of α seconds is applied to the unipolar potential signal corresponding to node ${\bar{s}}_{k}$, as shown in Equation (1). The design of bipolar EGMs following Equation (1) was addressed in the companion paper [13], and for this work, the configuration which reported better performance is chosen. Thus, the digital signal processing operator used as criterion to select the adjacent point ${\bar{s}}_{k}$ belonging to the neighborhood surrounding ${\bar{s}}_{i}$ is that of the maximum amplitude, i.e., choose the node which exhibits the signal with higher amplitude. In addition, the time delay value set to α is a time shift of 40 samples, which corresponds to approximately 20 ms for a sampling frequency of 2048 Hz.

Finally, the M-mode representation is extensively used throughout this paper because is a powerful tool to scrutinize time functions in a spatial domain. In the current study, the evidences attained with regard to the spatial–temporal distribution is based on their observation. The detailed definition and notation for the M-modes are developed in the companion paper. For reminding purposes, an M-mode is the representation of a set of functions, each one corresponding to one node of a surface mesh representing some part of the human body; the set of nodes is chosen to follow a pathway throughout the surface mesh. The results is a 3-D graph which reports visual information about the function waveforms across the selected path.

### 2.1. EGM Clustering

Clustering is one of the most used unsupervised learning methods during recent years in bioengineering [20,21,22,23]. The clustering problem consists of dividing a data set into several groups of elements so that the members of each group are as close as possible to each other, and the different groups are as different as possible (distant) to each other. This grouping aims to discover relationships not previously detected in a data set. Therefore, the result of applying a clustering technique to a data set is a set of clusters that provides us with partitions of said data set.

There are two types of clustering algorithms. In partitive clustering, groups are generated by methods that partition the data by creating a number *K* clusters. In general, these clustering methods use a distance measure to generate the clusters. In this kind of clustering, the most extended algorithm is *K*-means, which is used in this work and described in more detail later. There are other clustering algorithms such as density-based [24] or the self organizing maps [25], which are not considered here. In hierarchical clustering, a hierarchical structure of clusters is generated, which can follow a dividing approach [26] or an agglomerate approach [27]. Other types of clustering, such as the overlapping clustering grouping through fuzzy sets [28], or the probabilistic clustering, generating clusters from a probabilistic method as the Expectation–Maximization algorithm [29], could also be considered in future works.

A central question in clustering algorithms is to determine the similarity between objects by defining a distance measure. The purpose is to perform clusters of objects with high similarity. Thus, the resulting clustering will depend on the chosen distance measure, and it is not always possible to know which measure would be the optimal one in absolute terms.

The *K*-means clustering [30] is an iterative algorithm which separates *m* objects or observations, $x_{i}\in R^{n}$, i=1,⋯,m, into a partition of *K* clusters defined by their centroids $c_{k}\in R^{n}$, k=1,⋯,K. Each object belongs to the cluster with less distance with respect to its centroid: $\min_{k}d\left(x_{i},c_{k}\right)$. The choice of the number of clusters *K* leading to the greatest separation (distance) is not known a priori and it must be computed from the data. The objective of *K*-means clustering is to minimize the total intra-cluster variance. The algorithm takes two input parameters: the number of clusters, *K*, and the set of observations $x_{i}\in R^{n}$, i=1,⋯,m. Initially, a partition is chosen and the set of centroid values is initialized: $c_{1},c_{2},\cdots ,c_{K}\in R^{n}$. The algorithm consists of two steps:

1. Assign each observation to one cluster. For each observation $x_{i}$ (i=1,⋯,m) choose the index $l_{i}$ of the centroid closest to $x_{i}$:
  (2) $$l_{i}=\min_{k}d\left(x_{i},c_{k}\right),l_{i}=1,\cdots ,K$$
  where $l_{i}$ is the index of the cluster assigned to $x_{i}$.
2. Recalculate the centroid $c_{k}$ of the *k*-th cluster by averaging the points assigned to it.

These steps are repeated until either the centroid location no longer varies or the maximum number of iterations is attained.

As referred above, the choice of the distance affects the performance of the algorithm. Measures based on the $L_{2}$ norm (Euclidean distance) are commonly used:

(3) $$d_{L_{2}}\left(x_{i},c_{k}\right)={\left\|x_{i}-c_{k}\right\|}_{2}^{2}$$

For this case, the centroid of a cluster is calculated as the mean of the points which belong to that cluster. When $L_{1}$ norm (sum of absolute differences) is used:

(4) $$d_{L_{1}}\left(x_{i},c_{k}\right)={\left|x_{i}-c_{k}\right|}_{1}$$

each centroid are computed as the median of the points in that cluster. For the cosine distance:

(5) $$d_{c}\left(x_{i},c_{k}\right)=1-\frac{\langle x_{i},c_{k}\rangle }{{\left\|x_{i}\right\|}_{2}\cdot {\left\|c_{k}\right\|}_{2}}=1-\frac{x_{i}^{T}c_{k}}{\sqrt{\left(x_{i}^{T}x_{i}\right)}\sqrt{\left(c_{k}^{T}c_{k}\right)}}$$

the centroid is also calculated as the mean of the observations inside the cluster, after unit normalization of observations. Finally, for the correlation distance:

(6) $$d_{xc}\left(x_{i},c_{k}\right)=1-\frac{\langle \left(x_{i}-\bar{x}\right),\left(c_{k}-\bar{c}\right)\rangle }{{\left\|x_{i}-\bar{x}\right\|}_{2}\cdot {\left\|c_{k}-\bar{c}\right\|}_{2}}$$

each centroid is calculated as the mean of the points in that cluster over zero mean and unit norm observations. $\bar{x}$ and $\bar{c}$ stand for the observation and centroid means, respectively.

In *K*-means, the number of clusters has to be defined a priori, and the overall performance will depend on the centroids initialization. An appropriate choice for *K* can be achieved as the result of multiple runs, selecting the bets value for *K* according to a criterion defined according to the application requirements. In our study, different tests were performed for different *K* values and different distance measurements, which will allow us to reduce the error and not to fall into the data overfitting.

### 2.2. T-Wave Alternans Algorithms

TWA analyzes subtle fluctuations with the period of two heartbeats in the ventricular repolarization section of the ECG; it is manifested as variations in amplitude (very few μV) in the ST–T complex. TWA has brought considerable attention due to the scientific evidences of this phenomenon as sudden cardiac death risk stratifier [15]. Several detection/estimation methods have been developed, among which, three of the most popular ones are chosen for this work [16,31], namely: the Spectral Method (SM), the Modified Moving Average (MMA), and the Temporal Method (TM). Although, these methods were developed to be applied on the surface ECG, we use them on epicardial potentials in the same way as they are described in the literature, i.e., by processing the signal section corresponding to the ventricular repolarization; so either on surface potentials or on epicardial EGMs, the first step accomplished is the delineation of the ventricular repolarization segment, followed by its processing for signal conditioning purposes according to the accepted procedures in the field [31,32,33]: linear filtering, baseline drift removal, QRS detection, and delineation and alignment of the repolarization segment. Linear filtering is applied to the repolarization segment before alignment, and its purpose is to eliminate noise which falls outside the alternans bandwidth (frequency band [0.3,15] Hz), and it is implemented here as a simple lowpass filter with cutoff frequency set to be 15 Hz. Deviation of the baseline is cancelled through third-order spline interpolation. Upon QRS detection, the repolarization segment is extracted as a 300 ms length window beginning at a variable distance from the fiducial point, as described in [34]. Finally, the repolarization segment’s alignment is accomplished by maximizing the cross correlation of each segment with regard to a template, this one being obtained as the median of several repolarization segments taken from consecutive heartbeats [33].

To find whether the every-other-beat periodic pattern is present or not in the signal, *M* consecutive heartbeats are jointly processed. Therefore, let $x_{m}={\left[x_{m}\left(0\right),x_{m}\left(1\right),\cdots ,x_{m}\left(N-1\right)\right]}^{T}$ be the *N*-dimensional vector of samples corresponding to one single ventricular repolarization segment preprocessed as referred early. Subscript *m* stands for the *m*-th segment of a series of *M* extracted from consecutive heartbeats. Thus, the subset to be processed to find alternans can be enclosed in the following matrix:

(7) $$M={\left[x_{0}^{T},x_{1}^{T},\cdots ,x_{M-1}^{T}\right]}^{T}=[s_{0},s_{1},\cdots ,s_{N-1}]$$

where $s_{n}={\left[s_{n}\left(0\right),s_{n}\left(1\right),\cdots ,s_{n}\left(M-1\right)\right]}^{T}$ is the *n*-th beat series of a total of *N*.

Detection based on the SM consists of averaging the power spectral densities of the beat series $P_{n}\left(f\right)$:

(8) $$P\left(f\right)=\frac{1}{N}\sum_{n=0}^{N-1}P_{n}\left(f\right)$$

and assessing the *K*-score [14]

(9) $$K=\frac{P\left(0.5\right)-\mu_{noise}}{\sigma_{noise}}$$

where P(0.5) is the value of the aggregate spectrum (in Equation (8)) at the alternans frequency (0.5 cycles/beat); $\mu_{noise}$ and $\sigma_{noise}$ are the mean and the standard deviation of noise, estimated in a band close to 0.5 cycles/beat; *K*-scores values above 3 are considered as statistically significant, indicating alternans existence, as the measured alternans is at least three times over the level of uncertainty, as referred in [14]. An estimate of the alternant wave [16] is determined as $V_{alt}=\sqrt{P\left(0.5\right)-\mu_{noise}}$.

Following an alternative approach, the MMA [35] operates in the time domain by nonlinearly estimating the repolarization segments, as follows

(10) $${\hat{x}}_{m}={\hat{x}}_{m-2}+h_{m-2}$$

for m=2,3,⋯,M; ${\hat{x}}_{m}$ is the estimate of $x_{m}$, whose initial setting takes on the values: ${\hat{x}}_{0}=x_{0}$ and ${\hat{x}}_{1}=x_{1}$; array $h_{m}$ is a nonlinear correcting factor, calculated as explained in [16]. Notice that the MMA works over separated even and odd beats by computing the alternant wave as the difference between even and odd estimates $v_{l}={\hat{x}}_{2l-1}-{\hat{x}}_{2l-2}$, where $l=1,\cdots ,\left\lfloor \frac{M}{2}\right\rfloor$. The MMA is not a detector itself, but it is rather an alternant wave estimator, in which detection is performed through the maximum alternans magnitude,

(11) $$V_{l}=\max_{n}\left|v_{l}\right|$$

Another simpler, but effective, time domain method, is the so called TM [31], which is also an approach for estimating the TWA amplitude, which in this case is determined as:

(12) $$V_{alt}=\frac{1}{2}\max_{n}\left|{\bar{x}}_{odd}-{\bar{x}}_{even}\right|$$

where ${\bar{x}}_{even}=E\left\{x_{2l}\right\}$ and ${\bar{x}}_{odd}=E\left\{x_{2l+1}\right\}$, $l=0,1,\cdots ,\left\lfloor \frac{M}{2}\right\rfloor -1$, are the TWA templates for even and odd alternans, calculated as the sample mean (operator E stands for mathematical expectation).

## 3. Experiments and Results

In this section, we include the results of a set of experiments for each of the scrutinized EGM properties, namely, the spatial–temporal distribution in terms of the EGM waveform, and the spatial–temporal distribution of the repolarization properties in terms of the TWA algorithms proposed in the literature. We restrict ourselves to present evidence with intensive analysis of all the signals available in a few set of cases. We would like to stress that the focus of this work is not to provide at this point scientific evidence for the clinical application of the analyzed algorithms to the clinical practice, which would require devoted studies and trials, but rather to show that the ECGI recordings need to be analyzed with spatial–temporal based principles for digital signal processing. Therefore, rather than populational studies in large datasets, detailed explanations and analysis are herein given for each specific case. This is a usual approach sometimes followed in the ECGI literature, and the analysis of large datasets remains out of the main scope of the work.

### 3.1. EGM Clustering in the Presence of Infarction

We next describe the experiments performed to obtain a regionalization of epicardial cardiac tissue in terms of its EGM morphology. We use the unipolar EGM of some of the ECGI examples provided by Rudy’s laboratory and the bipolar EGMs obtained as explained in Section 2. First, the *K*-means unsupervised learning algorithm is used for the regionalization of cardiac tissue with unipolar EGMs from the ECGI system, and then, bipolar EGMs calculated with DSPO $\theta_{V_{\alpha }}$ are used to visualize the regionalization of the cardiac tissue. Both regionalizations are obtained with the *K*-means algorithm, and their comparison shows the similarity and compactness in the regionalization of the cardiac tissue provided by both methods. For any clustering problem, the *K*-means algorithm provides us with a regionalization of the multidimensional space represented by the available set of observed vectors, an identification of the centroid vector for each region, and the assignation of each observed vector to one of the regions according to some suitable distance between observed vector and centroid. Given that in this problem we have associated a spatial localization on the anatomical surface with the observed vectors, it is possible to create a map in the anatomical space indicating those observed signals corresponding to the same multidimensional regions in the signal space.

The preprocessing of each signal consisted on baseline wander cancellation, low-pass filtering, EGM-peak detection, and segmentation of 1.25 ms with said absolute peak at the 0.33 s in each signal. Only the first beat was considered, and no template was obtained.

**Clustering Results for Unipolar EGMs in the Presence of Infarction.** The input space of the *K*-means algorithm consisted first of the pre-processed unipolar EGMs, which had been removed from the baseline and for which a time window corresponding to a heartbeat has been selected. The regions that can be qualitatively and a priori distinguished in the cardiac tissue for the example of the infarction patient are scar region, border region, valve region, and healthy region. These regions can be differentiated according to the amplitude values used in electrophysiological studies for cardiac arrhythmia ablation. In the case of unipolar EGM, the values were: scar region ≤3 mV, border region between 3 mV and 5 mV, valve <3 mV, and healthy region ≥5 mV [36,37]. Several distances among beats in different locations were tested for different values of *K*, between K=3 and K=6, and spatial consistency was analyzed with respect to the unipolar EGMs of each node.

**Figure 1:**
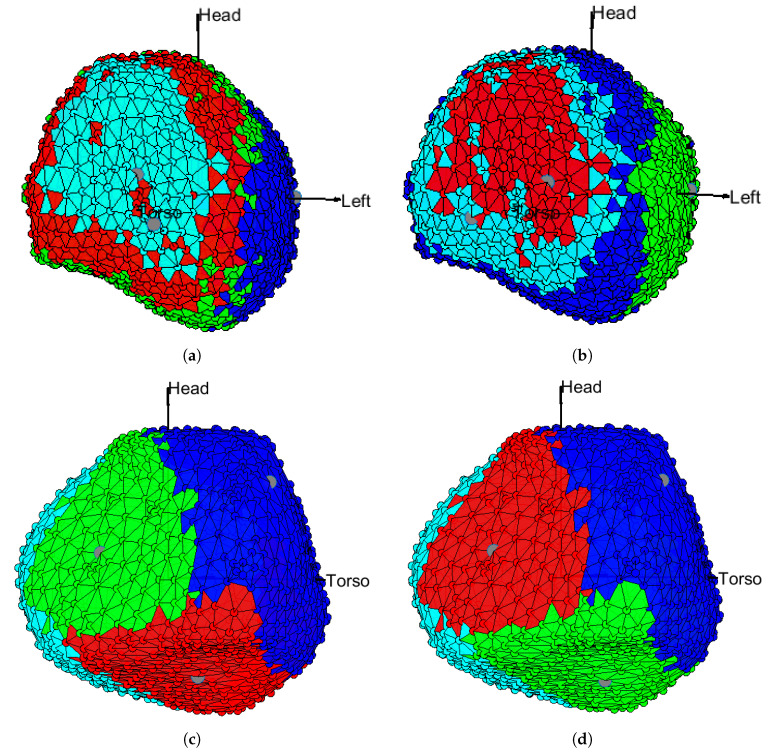
Unipolar Electrograms (EGM) in the presence of infarction. The distances used in the *K*-means algorithm with K=4 were: (**a**) $L_{1}$ distance; (**b**) Euclidean distance; (**c**) cosine distance; (**d**) correlation distance.

Figure 1 shows the regionalization provided by clustering for K=4 with all the tested distances. We can see that both $L_{1}$ and Euclidean distances give the worst regionalization for the cardiac tissue, as the clusters mix and exhibit unconnected and spatially inconsistent regions. On the other hand, the cosine and correlation distances provide a much more connected and compact regionalization of the cardiac tissue, which corresponds with the clinically and roughly established regions. The same experiment was checked in other cases showing similar behavior (not shown in this work). According to these results, the distances used for further testing in the remaining of this paper will be cosine and correlation distances.

**Unipolar EGM Clustering in the Presence of Infarction Using the Cosine and the Correlation Distances.** We next scrutinize whether any of these two distances are more convenient to regionalize the cardiac tissue from unipolar EGM, and the effect of *K* is analyzed again with detail in the infarction patient example.

**Figure 2:**
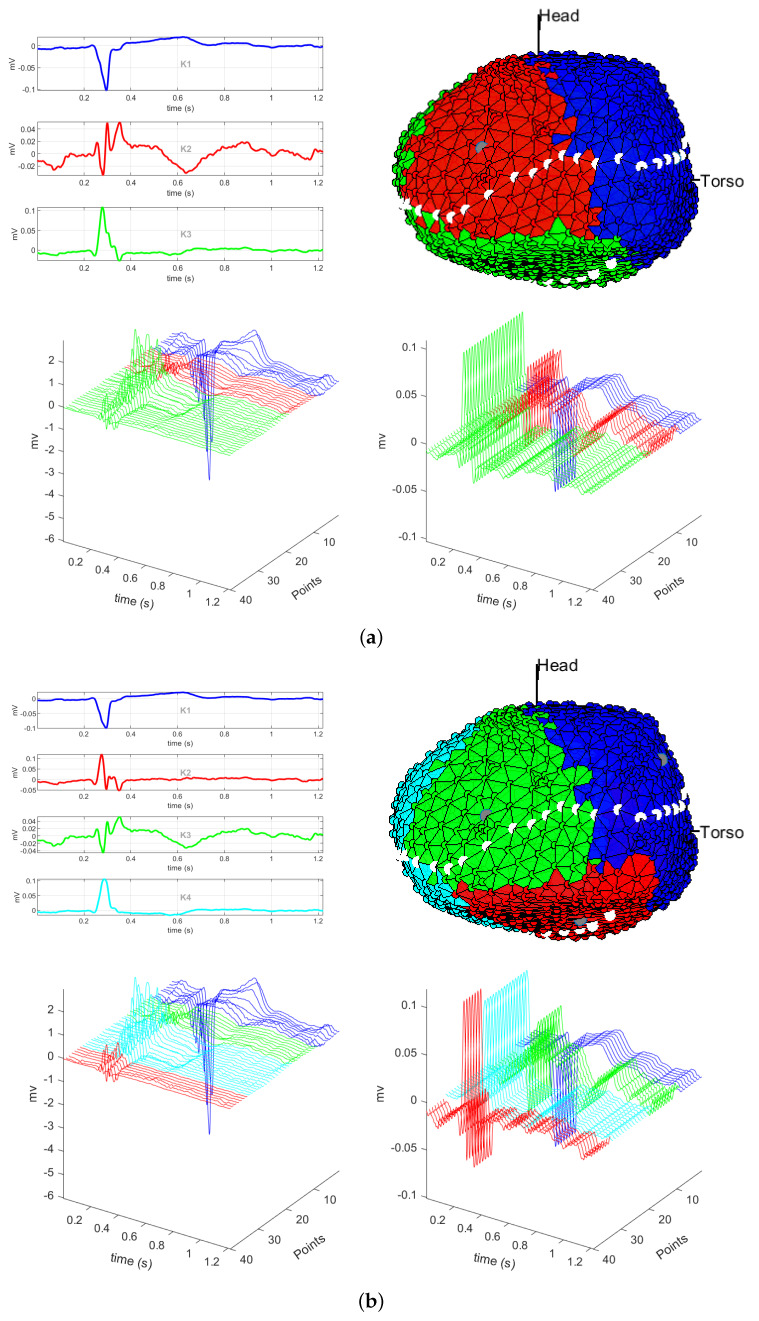
Unipolar EGM in the presence of infarction, using cosine distance with K=3 (**a**) and with k=4 (**b**). For each panel: (Up) centroids of each class and clustering map; (Down) M-mode of the unipolar EGM for the dotted line (left), and M-mode of their corresponding centroids (right).

**Figure 3:**
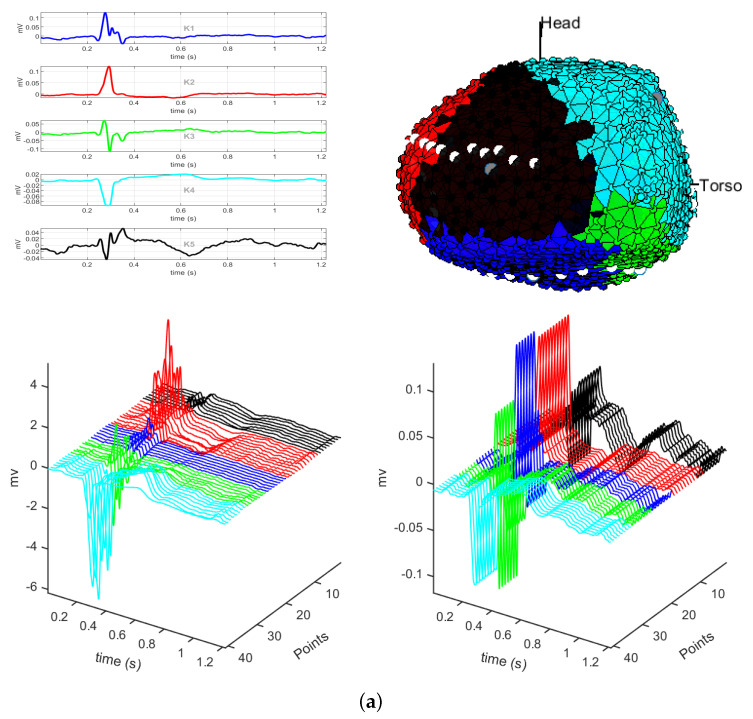

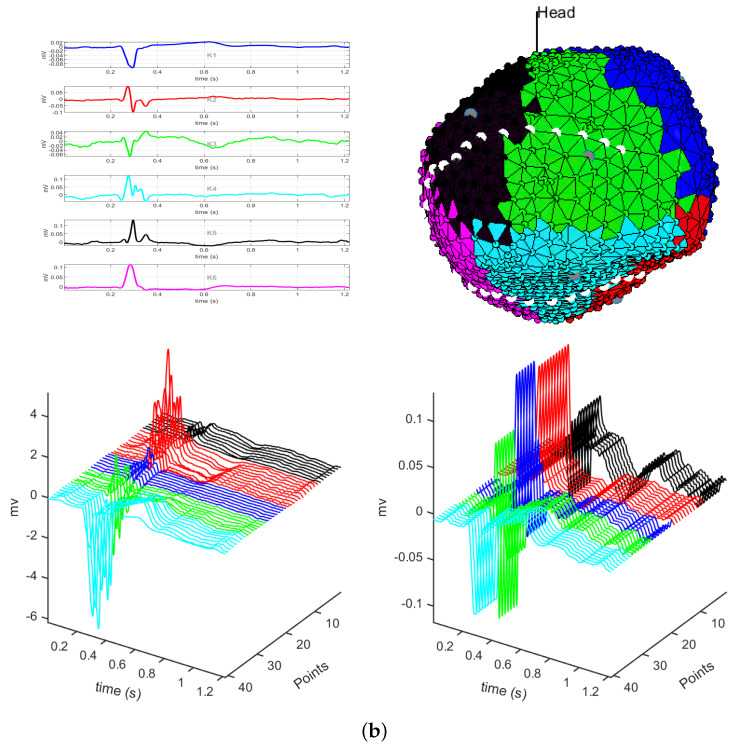
Unipolar EGM in the presence of infarction, using cosine distance with K=5 (**a**) and with k=6 (**b**). Continued from Figure 2.

Figure 2 and Figure 3 show the results for the cosine distance for several *K* values. Notice that both figures consist of four panels, numbered from (a) to (d), each for *K* values ranging from 3 to 6. Panel (a) shows the results of the *K*-means algorithm for K=3 when using the cosine distance. The upper left plot shows the selected centroids, with each class representing three regions of the cardiac tissue. These three centroids correspond to a unipolar EGM on the right side of the cardiac tissue, to a unipolar EGM of the valve region, and to a unipolar EGM on the left and inferior side of the cardiac tissue. The centroids are very different from one another, so the regionalization performed by the algorithm could be considered as acceptable. However, we can see in the color map that the class identified in the lower part of the cardiac tissue is the same as the one on the left side, and this regionalization is unlikely to be real, since it is precisely in the lower part of the cardiac tissue where we find the unipolar EGMs with smaller amplitude and greater fragmentation, as well as in the scar region of the cardiac tissue. We can check this in the M-modes of the lower plots of this panel, where the last unipolar EGMs of the green class correspond to the unipolar EGMs of the border or scar region of the cardiac tissue, given that they have amplitude lower than 4 mV and that they exhibit reduced fragmentation. Hence, these unipolar EGMs of the border region should correspond to a different class or should belong to the valve class, instead of their current assignment. For this reason, we consider that the number of clusters *K* chosen in this case for the *K*-means algorithm is not the most appropriate for EGM regionalization in this patient.

Panel (b), for K=4, shows the centroids of the four classes K1 up to K4. Classes K1 and K4 represent the unipolar EGMs in non-fragmented and healthy regions, which in the cluster map correspond to the left and right areas of the cardiac tissue. On the other hand, classes K2 and K3 represent fragmented unipolar EGMs, which are typical from the regions of the valve, border regions, and scar region, recalling that the last one is in the lower part of the cardiac tissue. In the clustering map, each class is compact, clearly separated from the others, and representing these four regions. In the M-modes, we can see the uniformity of unipolar EGMs of each class and the clear transition from one to another in the unipolar EGMs. In the case of classes K1 and K4, depicted in blue and light blue in the M-mode graph, respectively, the unipolar EGMs that are in the class boundary begin to be fragmented and to have low amplitude, which indicates that we are changing the region of the cardiac tissue, but these are morphologically similar to the rest of the unipolar EGMs of the class. On the other hand, the unipolar EGMs of classes K2 and K3, represented in red and green, respectively, are unipolar EGMs that present fragmentation as well as amplitude larger than 3 mV. According to this, the region represented by these classes are the valve, border regions, and scar region of the cardiac tissue. When observing the M-mode for these classes, we do not see a clear transition between classes, since the amplitudes and the morphology of these regions are mostly uniform, allowing us to identify very clearly each of those regions. Therefore, choosing K=4 for *K*-means algorithm, we can clearly see a grouping of the unipolar EGMs accounting for the presence of fragmentation, which can be checked in the centroids.

Panel (c) shows the results for K=5. It can be observed that centroids K2 and K4 represent unipolar EGMs with similar but inverted morphology, as represented in the clustering map, for the healthy region of the cardiac tissue corresponding to the left and right regions of the cardiac tissue. On the other hand, centroids K1, K3, and K5 belong to three different unipolar EGM classes where each of them is fragmented and in the clustering map we can identify them in the valve region. The lower one corresponds to the scar region of the cardiac tissue. Particularly, K1 and K3 are the classes that represent now the scar region of the cardiac tissue. If we analyze the M-modes, we see that unipolar EGMs in the transition from one class to another exhibit few differences in their morphology, but in terms of their amplitude, the unipolar EGMs of K3 class (green) have larger amplitude than the unipolar EGMs of K1 class (blue). This may be due to that K3 class is a border region between the scar and the healthy region, where some unipolar EGMs can be fragmented due to the existence of slow conduction paths typical of the border region of a scar region. In summary, the *K*-means algorithm with K=5 exhibits a good-quality regionalization of unipolar EGMs. Pretty much the same can be said with K=4, but *K*-means with 5 clusters enables the identification of some likely spurious border regions of the cardiac tissue.

Panel (d), for K=6, shows a spurious division of the valve region. This can be seen in the clustering map, where classes K5 and K3 correspond to the valve region. In the top left graph, we can see that these two classes correspond to fragmented unipolar EGM, but that they have different amplitudes. This is likely due to the presence of far field in that region and because it is a border region between the valve and healthy regions of the cardiac tissue. This can be better scrutinized in the M-mode, where the green unipolar EGMs, corresponding to K3 class, have smaller amplitude than the black unipolar EGMs, corresponding to K5 class.

As a conclusion of this subanalysis, by increasing the number of classes in the *K*-means algorithm, its regionalization accounts for the unipolar EGMs which exhibit increased fragmentation in their morphology, thus causing a spurious division of those areas. In particular, a consequence of this is a greater division of the scar and valve regions of the cardiac tissue. Values of K=4 and K=5 seem to be adequate for this description in the present patient. In other cases (i.e., other patients and other pathologies), the principles presented here can support the identification of the adequate number of regions.

**Figure 4:**
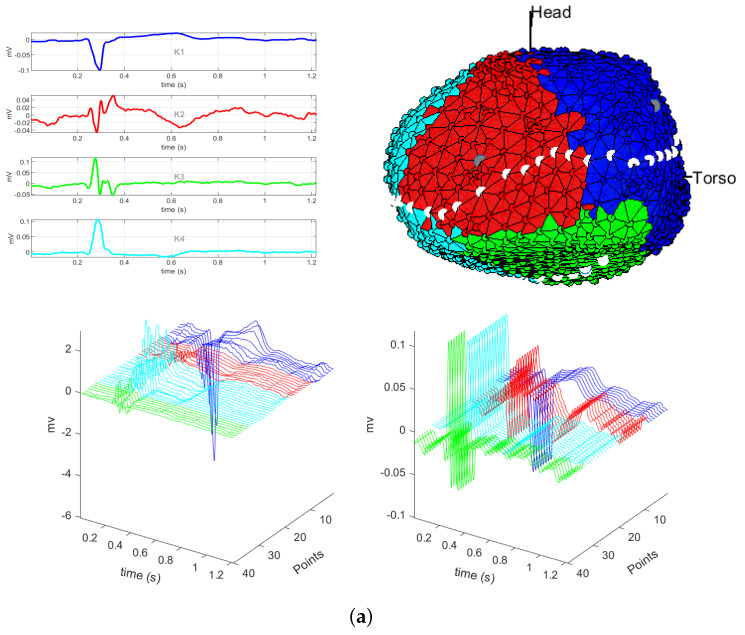

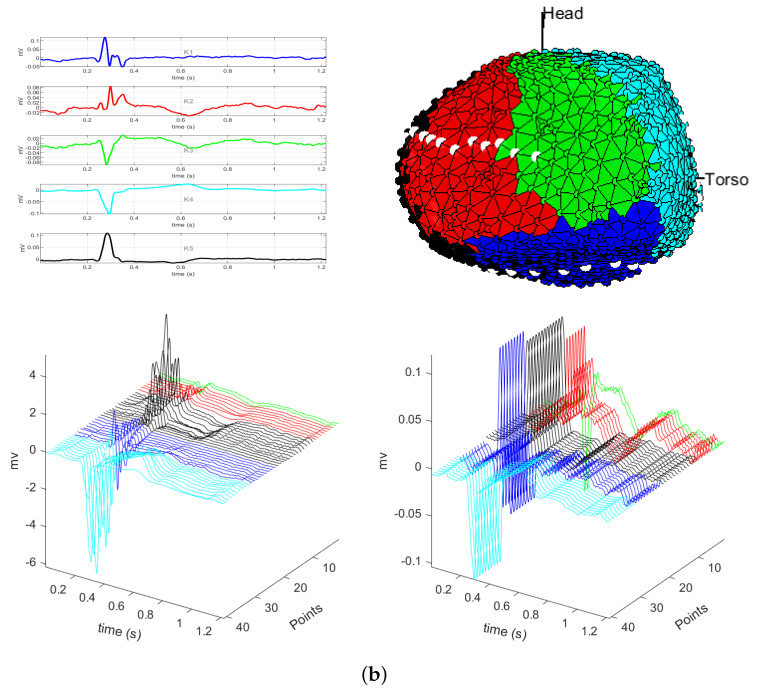
Unipolar EGM in the presence of infarction, using correlation distance with K=4 (**a**) and with k=5 (**b**). Panel contents are similar to the ones described in Figure 2 and Figure 3.

On the other hand, Figure 4 shows the results obtained for clustering when using the correlation distance and for different values of *K*. As with the previous distances, we can see that the most informative neighborhoods were K=4, K=5, and K=6 (not shown), so that for this distance we only analyze here two representative values. Panel (a) shows the classes obtained for K=4. For classes K1 and K2 (blue and red), we can see non-fragmented unipolar EGMs in the healthy region of the cardiac tissue. These classes can be seen in the clustering map (blue and light blue), one on the left and another on the right of the cardiac tissue, respectively. Classes K3 and K4 correspond to the scar and the valve region of the cardiac tissue. In these classes, the unipolar EGMs exhibit fragmentation and the morphology is very different, thus they are able to clearly identify each region. In the M-modes, we can see the unipolar EGMs of each of the regions of the cardiac tissue, where the unipolar EGMs of the edges are clearly differentiated between each class. On the other hand, unipolar EGMs of the scar region and valve region are clearly identified, because they exhibit fragmented unipolar EGMs and with amplitudes lower than 3 mV. In the case of K=5, see Panel (b), we can see that the valve region is divided into two classes, namely, K3 and K2. This classification should be uncommon, since this region has only highly fragmented unipolar EGM, and centroid K3 is a non-fragmented unipolar EGM waveform. The other classes correspond to the cardiac tissue regions. When analyzing the M-modes, we see spurious transitions between class boundaries, except between classes K3 and K2.

In general, both correlation and cosine distances present good regionalization capabilities of the cardiac tissue when using unipolar EGMs, identifying the regions of interest in the EPSs (scar region, border regions, and healthy region). With the correlation distance, K=4 is enough in this example to identify the regions of interest in the cardiac tissue, and each region is clearly delimited. On the other hand, the cosine distance with K=5 clearlier regionalizes the border regions, allowing good identification, which can be relevant in the EPSs since it is where scar regions begin. The clear identification of the border regions can be an advantageous information in EPSs, since it provides information regarding the location of the scar region of the cardiac tissue, therefore, a region where arrhythmia mechanisms can originate. With this information, electrophysiologists could guide and perform ablations of the cardiac tissue with greater precision, thus potentially decreasing the duration of EPSs.

**Bipolar EGM Clustering in the Presence of Infarction.** We next present a regionalization analysis of the cardiac tissue with the bipolar EGM obtained with the DSPO $\theta_{V_{\alpha }}$ proposed in the companion paper [13], for the example of an infarction patient. In this case, the input vector space of the *K*-means algorithm is given by the bipolar EGMs obtained with the $\theta_{V_{\alpha }}$ configuration, where the continuous-time shift α is set to be corresponding to 40 samples. As in the study for unipolar EGM, for the bipolar EGMs obtained by applying this operator, a time window corresponding to a heartbeat is selected. For the identification of the regions, the fragmentation and amplitude clinical criteria of bipolar EGMs are used. For this case, the amplitude of the bipolar EGMs for the scar region is less than 0.5 mV, the border region is between 0.5 and 1.5 mV, and the healthy region is greater than 1.5 mV [36,37].

For each distance method, K=5 was used to determine which of these methods provide better regionalization of the cardiac tissue. Note that in the previous study with the unipolar EGM, the regions of interest of the cardiac tissue were identified with K=4, however, with K=5, the border region of the cardiac tissue can be identified. That is the reason for which the number of clusters is set to be K=5. In the case of bipolar EGMs, we looked with greater interest for the identification of the border region in the cardiac tissue.

**Figure 5:**
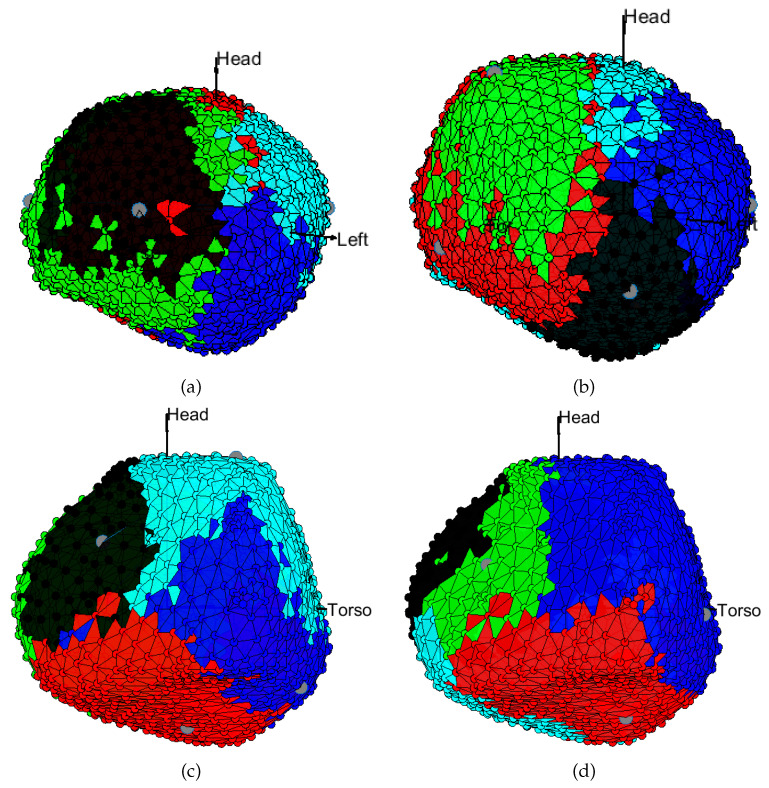
Bipolar EGMs in the presence of infarction. The distances used in the *K*-means algorithm with K=5 correspond to: (**a**) $L_{1}$ distance; (**b**) Euclidean distance; (**c**) cosine distance; (**d**) correlation distance.

Figure 5a,b depict the results of the *K*-means (K=5) with $L_{1}$ and $L_{2}$ norms, respectively. Notice that *K*-means algorithm does not separate well the regions of the cardiac tissue. In Figure 5a, regions, such as the left side of the cardiac tissue, present some ambiguity in its regionalization. In Figure 5b, the classes in the region of the septum are not very well determined. Although for both distances the valve and scar regions are well identified, other regions of the cardiac tissue exhibit a mixture of classes, and regionalization of the cardiac tissue is unclear. Figure 5c,d shows the results for the cosine and correlation distance methods, respectively. In Figure 5c, the regions of the classes are very well delimited, identifying well the regions of interest in EPS of the cardiac tissue; and in particular a border region is identified. In Figure 5d, the right lateral region is not well defined by the classes of the *K*-means algorithm, being the regions of interest difficult to identify. In both distance measurements, the valve region is differentiated from the scar region, providing a clear identification of these regions.

**Figure 6:**
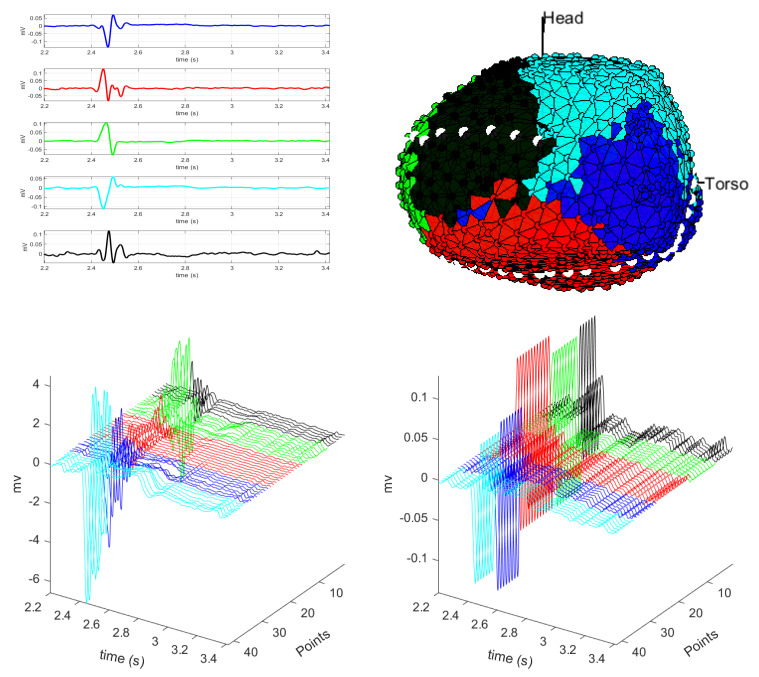
The cosine distance method with K=5. The upper left panel shows the centroids of each class and the right one, the clustering map. The lower left panel shows the M-mode of the bipolar EGMs of each region where the dotted line passes, and right one, depicts the M-mode of the centroids of the same line.

In general, we can see that the regions of interest for an EPS of the cardiac tissue are better identified when *K*-means clustering is used with the cosine distance, because it is capable of identifying a border region when the number of clusters is set to be K=5. According to these results, further analysis accomplished in this work are performed with K=5 and the cosine distance. Figure 6 shows the results obtained with these settings, and we can see the 5 classes yielded by the K-means algorithm. The upper panel shows the centroids of the 5 classes and the clustering map. Centroids K3 and K4 correspond to healthy regions of the cardiac tissue, and they also correspond to the left and right sides of the heart, respectively. Centroid K2 corresponds to the scar region, and centroid K5 corresponds to the valve region. Centroid K1 corresponds to the border region of the cardiac tissue, which lies between the scar region and the healthy region of the side left of the heart.

The lower panel of Figure 6 shows the M-modes of the dotted line, starting at the valve region and ending at the healthy region on the left side of the heart. The dotted line passes through the 5 classes that the *K*-means algorithm segments as the different regions of the cardiac tissue. In the shown M-mode, the first bipolar EGMs represent the valve region, followed by the green bipolar EGMs, which correspond to the healthy region of the cardiac tissue. The red bipolar EGMs are those of the scar region, which have a low amplitude and belong to the K2 class. Then we can identify the border region, corresponding to class K1, where we can see bipolar EGMs of amplitude between 0.5 and 1.5 mV, being the last ones with an amplitude greater than this range. Finally, we find that the bipolar EGMs in class K4 correspond to the healthy region of the cardiac tissue.

In this study, we can see that the unsupervised *K*-means algorithm can provide a reasonable regionalization of cardiac tissue using bipolar EGMs with the cosine method and K=5. These parameters are informative in order to distinguish border regions, with potential for guiding and supporting the electrophysiologist in the EPSs to locate scar regions of the cardiac tissue and improving the security in the location of the arrhythmia mechanisms. Nevertheless, further and devoted studies should develop and extend these findings.

**Figure 7:**
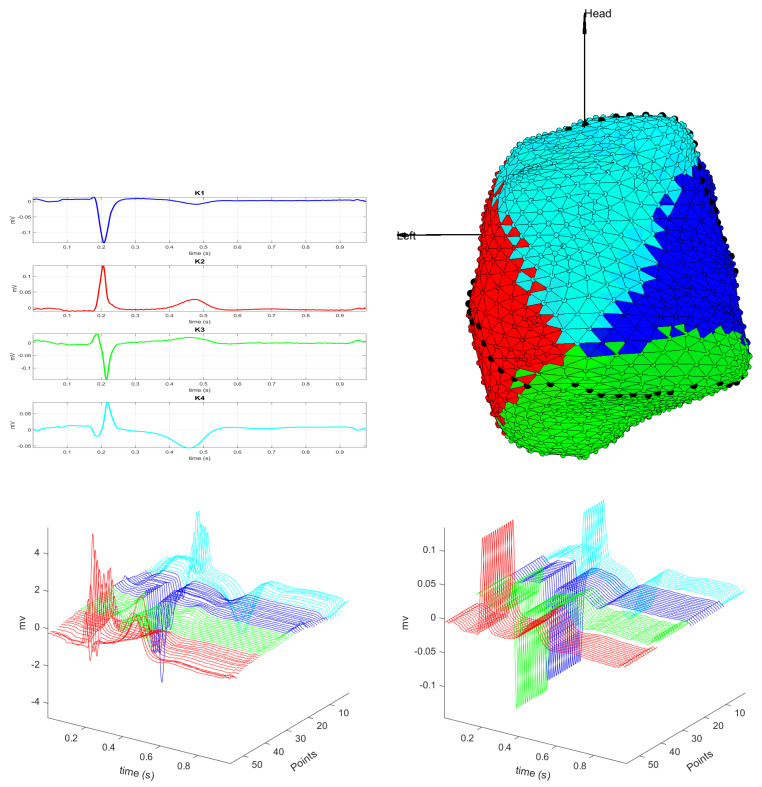
Control cases. Unipolar EGM, using cosine distance with K=4. (**Up**) Centroids of each class and clustering map. (**Down**) M-mode of the unipolar EGM for the dotted line, and M-mode of their corresponding centroids.

**Unipolar EGM Clustering in a Control Subject.**Figure 7 shows an example of clustering in a healthy control subject. We scrutinize only the case with K=4 classes, noting that in this case the identified regions are compact and well connected. Note that in this case the M-mode shows that region widths are long enough for not detecting any spurious region and to have the centroids as representative of those tissue zones. The smooth amplitude transitions between neighbor regions are well represented by the centroids, both when using the cosine distance (example in the figure) and when using the correlation distance (not shown). Note the positive and negative axis off the EGMs in opposite sides of the myocardium and also the progressive transitions in the repolarization waveform.

### 3.2. Comparison of TWA Algorithms

In this experiment, we conducted a detailed comparison of the estimation of TWA dynamics provided by the three explained algorithms. The same control subject and a LQTS patient were chosen to compare the information possibly retrieved by each of them. The details of the preprocessing within the framework of any of the TWA detectors are introduced in Section 2.2, and all these steps were applied to all the signals from each node. Regarding QRS detection, i.e., the time instant corresponding to ventricular depolarization, the local-maximum absolute potential peaks were detected with an adaptive threshold algorithm for each time signal. Similar tuning values were used for torso potentials and for epicardial potentials in both individuals, thanks to the amplitude-adaptive nature of the threshold in the peak detector. Each signal was beat segmented, according to their heart rate, and a time window of 300 ms after depolarization deflection was subsegmented in each beat, yielding the depolarization waveform. The even-odd matrices were built according to the repolarization equations described in Section 2.2. Each TWA estimation algorithm was applied to each signal, again with the same tuning parameters for torso and epicardium, and for both individuals.

**Figure 8:**
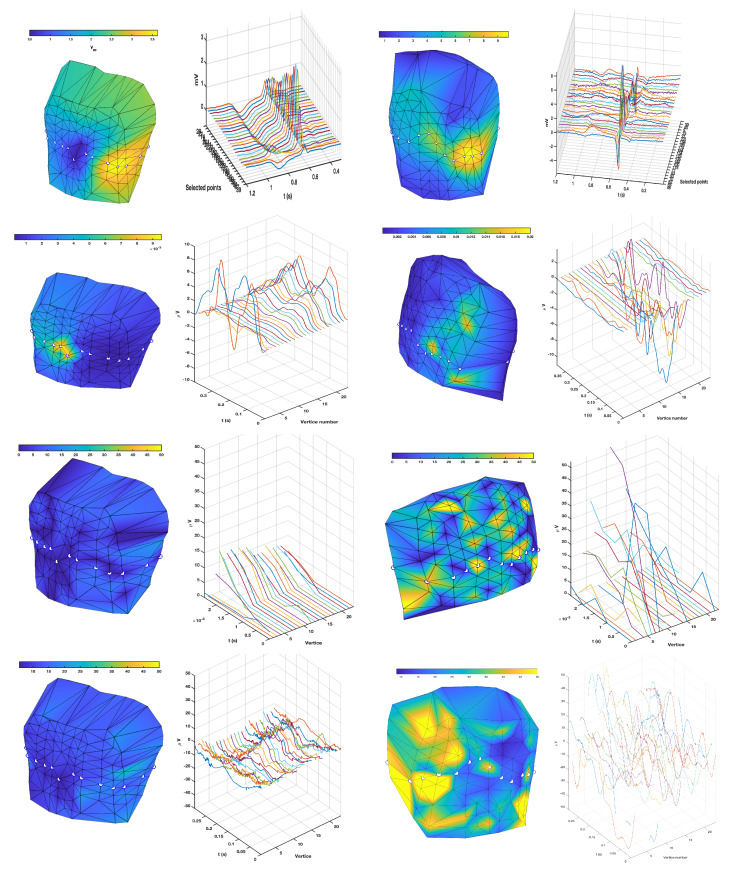
Analysis of T-wave alternans (TWA) detection algorithms in Torso recordings, for control subject (**left**) and Long-QT syndrome (LQTS) patient (**right**). The first row shows the voltage maps and M-mode for EGMs on the path in white points. The second, third, and fourth rows show the maximum amplitude maps and the TWA estimation provided by the temporal estimation, by the spectral method, and by the Modified Moving Average (MMA) method, respectively. The M-modes and colorbars are adapted to give a better understanding of the results. Note that these are not EGM potentials, but instead estimation of alternans amplitudes (in the temporal method) or of its measurement though some closely related index (spectral and MMA methods). The alternans amplitudes change spatially and temporally smooth in the control subject, whereas they exhibit noticeable spatial and temporal fluctuations in the LQTS patient. The line of points for the M-mode was selected to follow significant changes in the EGM amplitudes, and hence being able to check that the alternan’s changes in amplitude were not just an echo of the EGM changes in amplitude.

Figure 8 shows the results for the torso. The first row of panels shows the EGM amplitude maps, given by the peak-to-peak maximum amplitude of the first beat at each node on the torso. A representing path was marked for each individual on the torso (white points), following a trajectory to ensure the largest and the smallest EGMs amplitudes in each case, on a near-sagittal circuit, and the M-mode of the EGMs recorded on said points is represented for the complete beats, i.e., showing depolarization and repolarization. Amplitude maps were roughly similar in both individuals, though the relative lower amplitude in the breastbone zone compared to the higher amplitude in the apical zone was higher in the LQTS patient than in the control subject. The M-mode in the control subject showed smoothly aligned neighbor EGMs, with a well defined depolarization phase and a time-and-space sharp progressive repolarization phase. Regarding the patient, depolarization phase similarly exhibited smoothly aligned waveforms with neighbour nodes, but repolarization in this case was more heterogeneous in time and space, and with smaller amplitudes in general, compared to the amplitudes of its own depolarizations.

The next three rows of panels in the same figure show the maximum amplitude of the TWA estimation with each method, in both individuals (again left and right for control and LQTS), and the same path was used to describe the M-modes of local estimations in space and time. For the temporal methods, it can be observed that higher amplitude zones tended to be spatially grouped in the torso maps in both cases, and that these regions were in general uncorrelated with the EGM amplitude maps, hence ensuring that the TWA estimation is not an echo of the EGM amplitude. The M-modes of the spatial–temporal distributions had not much different amplitudes in general between both individuals, however, differences can be observed in the sense of variability and roughness, i.e., spatial–temporal changes in the estimated TWA morphology were smooth and progressive in the control individual, but randomized, non-smooth, and spatially uncorrelated in the LQTS signals. For the Spectral algorithm, the time evolution of the spectral parameter was represented for each node, as seen on the third row. In this case, it is highly evident both from the torso map and from the M-mode of the spectral TWA estimator that said estimator maximum amplitudes were markedly higher for the LQTS patient than for the control patient. The M-mode in this case again showed randomized and spatially uncorrelated activity for the LQTS patient, as well as smooth spatial correlation in the control subject. The fourth line of panels shows similar results for the MMA estimated alternans distribution, which again was with larger amplitude and spatially uncorrelated in the LQTS patient, and smaller amplitude and smoothly-changing in space and time for the control subject. In this case, the MMA method provided with a clearer marker than the Spectral method, and both provided a better marker than the temporal method.

**Figure 9:**
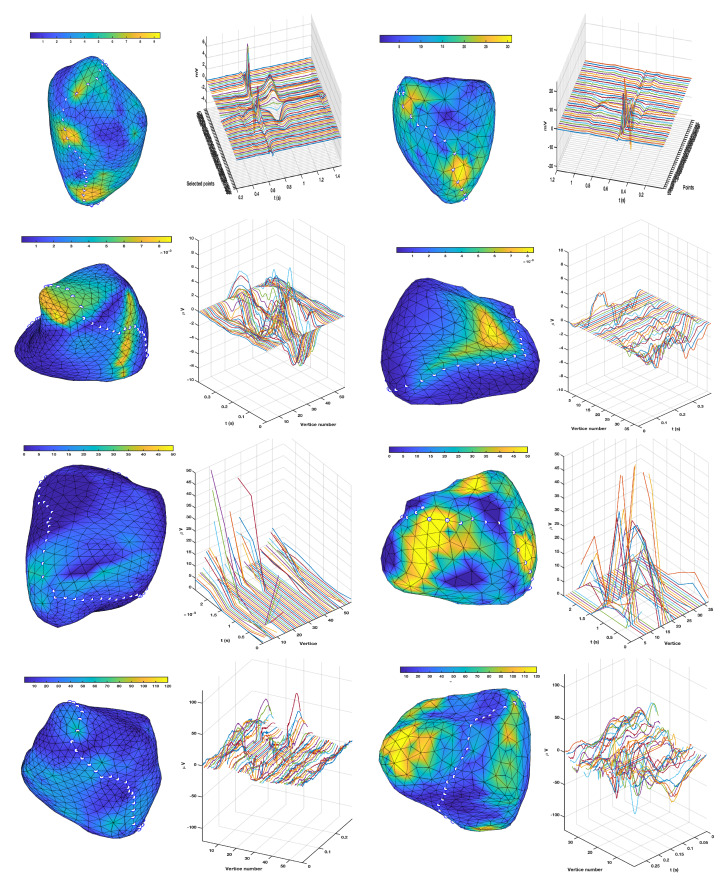
Analysis of TWA detection algorithms in epicardium recordings, for control subject (**left**) and LQTS patient (**right**). Content of panels is similar to Figure 8 but for epicardium instead of torso.

A similar analysis is shown for the TWA estimators on the epicardium, which are depicted in Figure 9. The EGM distribution shows that spatially local maxima are grouped in both cases (yellow regions). An M-mode was established following a path passing by several of said local maxima of EGM amplitude, to allow us to detect whether changes in the estimated TWA markers could be just attributed to the EGM amplitude, which can be checked that it was not the case. The repolarization spatial–temporal distribution was more visible in the control subject than in the LQTS patient, however, in the last one, this time the spatial–temporal local correlations were smoother and changes well localized in both dimensions, very differently from the repolarization potential distributions in the LQTS torso.

The TWA estimations from the temporal method yielded similar results to the torso, in the sense that estimated TWA waveforms were more similar in neighbor nodes for the control subject, whereas more irregularity was observed in the spatial–temporal LQTS patient. The maximum amplitude of this marker was not different in general from one to another subject. Note that in this case the M-modes could seem to be saying that few differences are observed, but this is an effect of the pathway chosen to support their representation so that the effect of the maximum EGM amplitude can be discarded as a biasing cause. According to the epicardium maps, Spectral method and MMA method again yielded very different amplitude distributions in the LQTS compared to the control subject, and the maximum amplitude regions are larger regions seen in yellow, and with some spatial coincidence between both methods.

## 4. Discussion and Conclusions

We have analyzed with detail several examples of complete ECGI recordings, and we have paid attention to the spatial–temporal properties of the bioelectric potentials recorded on the torso and on the epicardium, the last ones thanks to their inverse-problem estimation provided by currently existing techniques. Whereas the companion paper [13] focused on several implications of spatial–temporal preprocessing and calculation of bipolar potentials, in this one we focused on the spatial–temporal properties of the biopotentials waveform, using a beat-based clustering approach, and on the repolarization properties, using the TWA estimators commonly used in the literature for risk stratification with ECG recordings.

Our results with EGM clustering techniques shown that unipolar potentials estimated on the epicardium change smoothly with distance, which is consistent with the cosine distance and with the correlation distance being more suitable in this setting for regionalizing the epicardium morphology of the analyzed waveforms. Whereas the number of neighbors has an impact on the quality of the clustering results, the analyzed data have provided with an easy estimation of the number of appropriate clusters in different examples. Orders around K=4 or K=5 tend to give a compact set of regions, both in healthy conditions or in the presence of infarcted tissue. Lower orders group together signals with very different electrophysiological shapes, whereas higher orders tend to identify boundaries between neighbor regions as additional regions themselves, but also to detect some over-segmentation on the tissue. These findings are very basic from a signal processing and from a machine learning viewpoint, but show that simple algorithms accounting for spatial information can be useful. Whereas this holds for ECGI systems, where epicardial EGM estimations are provided for a variety of nodes, its translation to current intracardiac navigation systems could be not so straightforward, specially for those ones based on sequential data acquisition, as they usually require additional quality control in terms of the recorded EGMs even with recent multi-catheter electrodes [38,39,40].

Our results with repolarization analysis in terms of TWA estimators have thrown new ideas on the possible nature of the alternans. Three different estimation algorithms have been proposed, namely, the temporal method, the spectral method, and the MMA method. Their application on the torso has shown that the temporal method can give some information about altered repolarization in terms of estimated alternans waveform irregularity, rather than in terms of alternans amplitude. On the other hand, both spectral method and MMA method provide differences on the torso analysis both in terms of maximum amplitude and on irregularity, being more patent in the LQTS case example. When applied to the epicardial ECGI-estimated potentials, the temporal method still exhibits limitations in terms of amplitude, though spatial changes are more random. In addition, both properties (amplitude and spatial–temporal randomness) are markedly different when obtained in LQTS and in control cases, but also the maximum estimated TWA amplitude markers tend to be regionalized in some few regions which exhibit spatial consistency between methods.

We should note that the EGM on the valves are not of clinical interest, given that they correspond to far-field potentials, rather than to actual bioielectric activation on the substrate. However, the ECGI estimation system provides the estimated potentials in that region at the end of the inversion procedure. Whereas it would be immediate to exclude this region from subsequent analysis (as it is done in most works in this field), we decided to include them as they are low-amplitude and different-substrated biopotentials, so it is convenient for the purpose of showing the capabilities of different metrics in clustering at identifying different and related anatomical regions. Note then that the low amplitude due to far field is physiologically different from the low amplitude in slow-conducting regions. Note also that the thresholds that we have used here are disease-specific, according to the references provided by our cardiologist coauthor, and they refer to electrophysiogical criteria for infarcted tissue. Given that the amplitude of ECGI-estimated EGMs with currently available techniques could be suffering some distortion due to the regularization processes involved in their estimation, further work should be addressed before using these same threshold criteria on ECGI-estimated EGMs on patient populations.

Non-invasive cardiac mapping, or ECGI, is a novel, painless and relatively inexpensive method to map the patterns of electrical activation and repolarization of the heart, providing a valuable tool for early identification and diagnosis of abnormalities of conduction and arrhythmias. This is being used to yield a priori information to guide invasive surgical procedures, improve success rates, and reduce procedure time. This reconstruction from surface potentials needs to be complemented by the patient’s specific torso-cardiac geometry, which could fill the gap between the non-invasive 12-lead ECG (low resolution) and invasive electrophysiology studies (high resolution). Given the actual development of said systems, the use of atomic signal processing techniques should be revisited and reformulated, in order to account for spatial and temporal joint information, and several works are raising in this direction in the very recent literature. In [41], the technical details, clinical applications and current limitations of the methods commonly used in ECGI are collected to accelerate diagnosis, to guide therapy and improve risk stratification, and in subsequent work [42] the authors propose best practices for technical validation. In [43] the capabilities that an ideal cardiac mapping system should have are reviewed. The spatial–temporal information is often present in these works, but it is rarely mentioned explicitly.

The effects of signal processing on the inverse problem have been previously scrutinized, for instance, in [44], where three different sets of preprocessing operations were considered, namely, high-frequency noise removal, baseline drift removal, and signal averaging. For different cases in experimental data from a Lagerdorff-perfused pig heart within a human-shaped torso tank, the inverse problem was solved and reconstructed signals were compared with those actually recorded on the heart. Baseline drift and high-frequency noise dramatically affected the reconstructed EGM amplitude and the latency maps, respectively. It would have been interesting and informative to scrutinize the spatial–temporal distribution of the residuals for each of the scrutinized preprocessing stages. In addition, clustering techniques have been previously proposed to analyze intracardiac EGM. In [45], the identification of different spatial domains in terms of their fragmentation properties were scrutinized, using a semi-supervised, feature-based scheme for complex EGMs recorded during atrial fibrillation in intracardiac navigation systems. The main aim of this work was to identify ablation targets for atrial fibrillation. Atrial map distribution of different features were documented, though few attention was paid to the spatial distribution of the EGMs themselves, mostly due to this is an arrhythmia with very changing EGM waveforms. Nevertheless, few works can be found in the literature aiming to cluster the intracardiac EGM to identify different domains. An interesting precedent for characterizing the spatial distribution of TWA on the heart surface from ECGI measurements can be found in [46], where the authors developed an inverse spectral and it was applied on a canine model. The method unifies the equations of the inverse problem and of the estimated TWA with expected biphasic behavior, and interesting results are reported from the experimental model. Nevertheless, moderate insight is provided with respect to the TWA markers obtained in different regions, and few results are documented with respect to the apparent random-like (more than biphasic) changes in the spatial–temporal domain.

The main of the limitations of our study is that we focused on few case examples (one control, one infarction patient, and one LQTS patient). This was motivated by our main purpose here being to analyze with detail in said few cases the implications of different strategies for considering the spatial–temporal signal processing analysis and its possible repercussion on the possibilities offered by ECGI to the arrhythmia diagnosis and treatment. Each of the proposed spatial–temporal analysis in these two companion papers represents itself a direction to scrutinize with more detail and with larger datasets (ECGI preprocessing, bipolar EGMs from ECGI, clustering of ECGI potentials, and TWA markers from ECGI recordings). In addition, we restricted ourselves to use very simple signal processing and machine learning tools, whereas the results encourage the design and development of new analysis methods with a spatial–temporal bases in their conception.

ECGI is a non-invasive method that is increasingly allowing to map the electrical activity of the heart in humans, in real conditions, proposed and advanced by Rudy and his Team for years [47]. Today, reviews and summaries can be found on the results of ECGI studies of arrhythmogenic substrates associated with clinical arrhythmias such as heart failure, myocardial infarction, atrial fibrillation, and abnormal ventricular repolarization. The consideration of spatial–temporal signal analysis will undoubtedly bring more advance and enhanced scope to the possibilities of this modality which is the only one allowing us to visualize the biopotentials in the heart.
